# Translation and cultural adaptation of the hyperacusis handicap questionnaire to Brazilian Portuguese

**DOI:** 10.1590/2317-1782/e20250057en

**Published:** 2026-01-30

**Authors:** Adriane da Silva Assis, Stela Maris Aguiar Lemos, Prashanth Prabhu, Patrícia Cotta Mancini

**Affiliations:** 1 Programa de Pós-graduação em Ciências Fonoaudiológicas, Faculdade de Medicina, Universidade Federal de Minas Gerais – UFMG - Belo Horizonte (MG), Brasil.; 2 Programa de Pós-graduação em Ciências Fonoaudiológicas, Departamento de Fonoaudiologia, Faculdade de Medicina, Universidade Federal de Minas Gerais – UFMG - Belo Horizonte (MG), Brasil.; 3 Department of Audiology, All India Institute of Speech and Hearing - Mysore (Karnataka), India.

**Keywords:** Hyperacusis, Indicators of Quality of Life, Self-assessment, Surveys and Questionnaires, Translating, Speech, Language and Hearing Sciences

## Abstract

**Purpose:**

To translate and culturally adapt the Hyperacusis Handicap Questionnaire (HHQ) to Brazilian Portuguese.

**Methods:**

The cultural adaptation followed the Consensus-based Standards for the Selection of Health Measurement Instruments (COSMIN) guidelines and included five stages: 1) translation of the questionnaire from English to Brazilian Portuguese (BP) by two bilingual translators; 2) synthesis of the translations into a consensus version; 3) back-translation into English by two professional translators; 4) development of the pre-final version of the HHQ in Brazilian Portuguese by a committee of experts; and 5) testing of the pre-final and adjusted pre-final versions with individuals from the target population.

**Results:**

No discrepancies were observed in the title, key responses, or questionnaire items throughout the translation process. In the pre-final version testing phase, more than 80% of participants understood the HHQ items in BP, demonstrating the effectiveness of the adaptation. However, four items were adjusted based on participants’ suggestions during the testing phase. Subsequently, the adjusted pre-final version was tested, with 100% of participants demonstrating comprehension of the items, confirming its appropriateness for the Brazilian population. During the linguistic equivalence assessment, none of the participants selected the "not applicable" option, further validating the relevance of the questions to the Brazilian context. Additionally, an introductory statement was added, providing detailed instructions on how to complete the questionnaire.

**Conclusion:**

The cultural adaptation of the HHQ to Brazilian Portuguese was completed, resulting in the final version titled Hyperacusis Handicap Questionnaire in Brazilian Portuguese (HHQ-BP). Validation of this version is currently in progress.

## INTRODUCTION

Hyperacusis is an auditory condition characterized by an exaggerated sensitivity to sounds^(1)^. Unlike hearing loss, associated with difficulty detecting sounds, hyperacusis involves an amplified and intensified auditory processing of sound information. This condition causes everyday sounds, typically well-tolerated by most people, to become uncomfortable and even distressing for those affected^(2-3)^.

Studies estimate that the prevalence of hyperacusis in the general population ranges from approximately 8% to 17%^(4)^, while among children, this rate is around 3.7%^(5)^. In Brazil, specific data on the prevalence of hyperacusis in the general population is limited. However, a study conducted in a Brazilian town with 506 children aged 5 to 12 years reported a prevalence of 3.2%^(6)^. Notably, more than 90% of individuals with hyperacusis also report tinnitus^(7)^, and between 40% and 55% of people with tinnitus exhibit hyperacusis^(8,9)^. These findings suggest a complex interrelationship between these conditions, possibly indicating a common origin for both symptoms.

Hyperacusis can be classified into four subtypes^(1,2)^: (1) Loudness Hyperacusis: Occurs when moderately loud sounds are perceived as excessively intense, such as the sound of stacked plates. (2) Annoyance Hyperacusis (Misophonia): Characterized by irritation or panic triggered by specific sounds, even when they are not loud, such as chewing. (3) Fear Hyperacusis (Phonophobia): Involves an exaggerated fear of anticipated sounds, such as balloon popping. (4) Pain Hyperacusis: This manifests as physical pain in the ears or head in response to moderately intense sounds, such as alarms. These subtypes may present individually or in various combinations ^(10)^.

Although there is no cure for hyperacusis, several therapeutic approaches can be used, typically within a multidisciplinary model^(8,11)^. Treatment selection should be personalized, considering the severity of symptoms and their impact on quality of life^(8)^. For some patients, counseling may suffice; however, those with more severe symptoms may require psychological support to modify emotional responses to sounds and develop coping strategies^(8,12)^. When hyperacusis is a symptom of a specific health condition, it should be addressed alongside that condition. In cases where the cause is unknown, sound desensitization therapy has proven to be an effective option, helping patients gradually adapt to everyday sounds^(8)^.

Given the multifactorial nature of hyperacusis, applying questionnaires to assess the quality of life of affected individuals is essential. These tools allow for the identification of patients' specific needs, evaluation of the condition’s impact on daily activities, and improvement in clinical care, especially in auditory rehabilitation contexts where hearing aid adaptation is often performed.

Several questionnaires have been developed and validated to assess hyperacusis. These include the Hyperacusis Questionnaire (HQ)^(13)^,which measures and quantifies this condition; the German Questionnaire on Hypersensitivity to Sound (GÜF)^(14)^, which evaluates the subjective impact of hyperacusis and it is widely used in patients with tinnitus; the Multiple Activities Scale of Hyperacusis (MASH)^(15)^, which quantifies discomfort caused by sound in daily activities; the Sound Tolerance Interview and Questionnaire Instrument (STIQI)^(16)^, designed for hearing aid users, which investigates factors related to sound intolerance; the Inventory of Hyperacusis Symptoms (IHS)^(17)^, which enables the differentiation of hyperacusis subtypes; the Hyperacusis Impact Questionnaire (HIQ)^(18)^, which assesses the impact of hyperacusis on an individual's daily life; and the Hyperacusis Handicap Questionnaire (HHQ)^(19)^, which stands out for its structure encompassing three domains – functional, social, and emotional – providing a comprehensive evaluation of how hyperacusis interferes with quality of life, identifying the most affected areas, and supporting the prioritization of individualized management strategies.

In Brazil, the only validated questionnaire available for use with individuals with hyperacusis is the Hyperacusis Questionnaire (HQ)^(20)^, which aims to quantify and measure the severity of the condition. However, there is currently no Portuguese-language tool specifically designed to evaluate the effects of hyperacusis on quality of life.

In this context, the present study aims to translate and culturally adapt the Hyperacusis Handicap Questionnaire (HHQ)^(19)^ into Brazilian Portuguese. The HHQ was originally developed and validated in English in 2020 with a sample of 77 individuals presenting hyperacusis associated with tinnitus, including 41 males and 36 females, aged between 20 and 55 years. The questionnaire consists of 21 items covering three dimensions: functional, social, and emotional. Its purpose is to measure the severity of symptoms and the degree of disability associated with hyperacusis. The dimension-based scoring allows healthcare professionals to prioritize the most affected areas of quality of life during counseling, promoting treatment adherence and better clinical outcomes^(21)^.

The motivation for this research arises from clinical practice, where many patients seeking hearing aid adaptation report sound hypersensitivity, which may compromise acceptance and efficacy in auditory rehabilitation. Providing a tool capable of measuring the impact of hyperacusis on quality of life can enhance clinical assessment and support the development of more effective and personalized interventions, improving care and the quality of life for individuals affected by this condition.

## METHODS

This methodological study was conducted between June and November 2022, and approved by the Research Ethics Committee of Universidade Federal de Minas Gerais (UFMG) under approval number 5.809.522. All participants signed an informed consent form.

The Hyperacusis Handicap Questionnaire is an instrument to measure symptom severity and the degree of disability caused by sound hypersensitivity. It is a self-administered instrument consisting of 21 questions, with responses following a frequency scale: “never” (0 points), “sometimes” (2 points), and “always” (4 points). The questionnaire provides a total score as well as specific results for three dimensions: functional (questions 1-7), social (questions 8-14), and emotional (questions 15-21).

The translation of the HHQ questionnaire into Brazilian Portuguese was formally authorized by its original author via email correspondence. The translation and cultural adaptation of the HHQ into Brazilian Portuguese (BP) were conducted rigorously following the criteria established by the Consensus-based Standards for the selection of health Measurement Instruments (COSMIN)^(22),^ through the steps described below:

1. Translation: The original English questionnaire was independently translated into Brazilian Portuguese by two fluent translators, whose native language was Portuguese. One translator (T1) had no familiarity with the subject and was from the education field, while the other translator (T2) was a specialist in the area. The process included translating the questionnaire items, response options, and scoring instructions. The primary goal was to ensure conceptual equivalence while remaining faithful to the original meaning.
2. Synthesis of Translations: Two Brazilian healthcare professionals proficient in English, along with the two translators from the previous step, consolidated the two translations into a consensus version (T1-2). Linguistic and cultural adaptations were made to tailor the content to the Brazilian context while preserving the original meaning of the phrases. This step was performed based on T1, T2, and the original HHQ version.
3. Back Translation: The consensus version (T1-2) was back-translated into English (BT) by two independent professional translators, one a native North American and the other Brazilian, neither of whom had prior knowledge of the original questionnaire, ensuring impartiality. The two resulting versions (BT1 and BT2) were compared to assess fidelity to the original content.
4. Preparation of the Pre-Final Version: A committee of experts, including the translators, study authors, and a healthcare professional, reviewed all translations (T1, T2, T1-2, BT1, and BT2) to create the pre-final version (V1) of the HHQ. The analysis was based on semantic, idiomatic, conceptual, and cultural equivalence criteria^(23)^, comparing the original, translated, and back-translated versions. As a result, the pre-final version (V1) of the HHQ in Brazilian Portuguese was structured, including a formal statement with detailed instructions for completing the questionnaire.
5. Testing the Pre-Final Version (T-V1): V1 was administered to 30 individuals from the target population to evaluate the clarity, appropriateness, and applicability of the items. According to COSMIN guidelines^(22)^, this stage should include 30 to 40 participants representative of the target population. Therefore, the inclusion of 30 individuals with hyperacusis complies with international recommendations and is considered sufficient to identify potential issues related to item comprehension, as well as cultural and linguistic inadequacies. This step also allows for any necessary adjustments to the questionnaire before it is applied to a larger sample.

This step is crucial to ensure that participants fully understand the items without difficulty. To this end, a question regarding the clarity of each item was included, allowing for suggestions for improvement. An item was considered adequate if over 80% of participants reported no comprehension difficulties^(22)^. Otherwise, the item would be revised and retested. Additionally, a “not applicable” response option was added for items that did not apply to the target culture.

**Figure 1:**
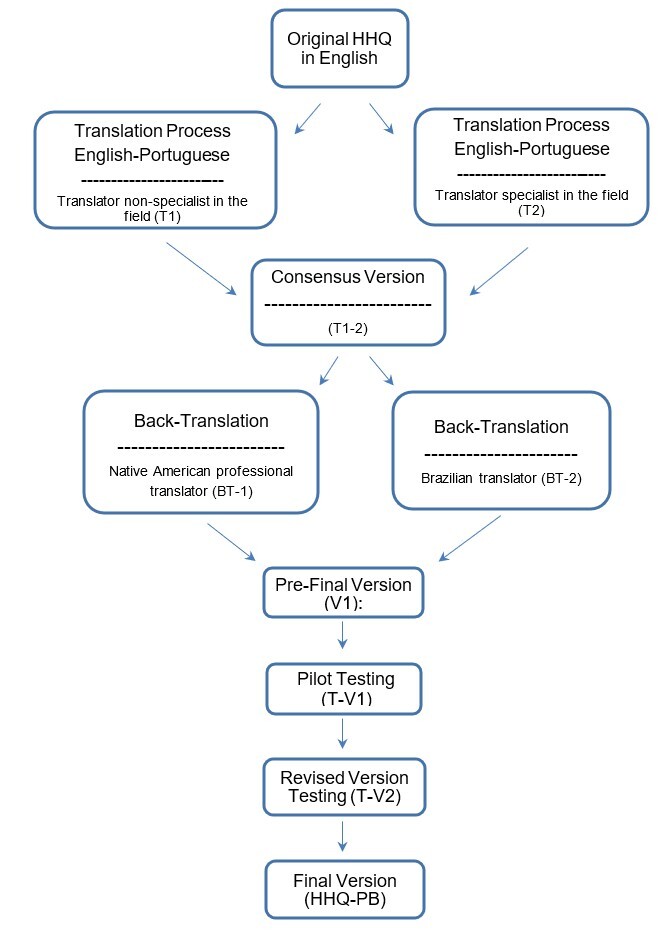
Translation and cultural adaptation process of the Hyperacusis Handicap Questionnaire to Brazilian Portuguese

The expert committee from the previous step analyzed the responses, finding that over 80% of participants reported no difficulty understanding the questionnaire items. Items 1, 4, 8, and 12 showed a comprehension rate close to 85%. After revisions based on participant suggestions, an adjusted version (V2) was developed, and a new test (T-V2) was conducted with another 30 participants. In this second round, 100% of participants reported understanding all items. Thus, the final version of the HHQ in Brazilian Portuguese (HHQ-BP) was established (Appendix A), adhering strictly to methodological and cultural criteria to ensure its applicability to the Brazilian population. The flowchart of the process steps is presented in Figure 1.

Data collection for the T-V1 and T-V2 phases was conducted at the Audiology Service, annex Hospital das Clínicas da UFMG, in the city of Belo Horizonte, Minas Gerais, Brazil. Patients reporting hyperacusis who attended basic audiological evaluations or were waiting for consultations were invited to participate in the study.

The inclusion criteria were as follows: individuals aged 18 to 70 years; of both sexes; diagnosed with hyperacusis; Brazilian nationality; literacy in Portuguese; and audiological test results within normal limits, as per the standards of Lloyd and Kaplan^(24)^. Participants were excluded if they had neurological, cognitive, and/or psychiatric conditions (evident or self-reported), a score below the expected level on the Mini-Mental State Examination for their education level^(25)^, or demonstrated difficulty completing the questionnaire or proposed evaluations.

To assess the participants' hearing, pure tone threshold (PTTs) and tympanometry were used. Initially, meatoscopy was performed to ensure that the external auditory canal was in suitable condition for subsequent testing.

Pure tone threshold testing was conducted in an acoustic booth. Participants were instructed to raise their hand upon hearing a tone, even if it was very soft. Air conduction thresholds were measured at frequencies ranging from 250 to 8000 Hz. Normal hearing was defined as thresholds below 25 dB HL, according to the criteria established by Lloyd and Kaplan^(24)^.

Tympanometry was performed to assess the functional integrity of middle ear structures, following the criteria proposed by Jerger^(26)^.

The Loudness Discomfort Level (LDL) was assessed after audiometric thresholds were obtained, using pure tones at 500, 1,000, 2,000, 3,000, and 4,000 Hz in both ears. The stimulus was initially presented at 50 dB HL, and its intensity was increased in 5 dB steps. Each tone was presented for two seconds, with a one-second interval between presentations, until the participant reported discomfort. The level at which discomfort obtained was reported was recorded as the discomfort threshold for that frequency^(27)^.

The instructions provided to participants were standardized to ensure consistency in responses. All participants received the following instructions: "You will hear tones that will gradually get louder. Please raise your hand when the sound reaches an intensity that you no longer wish to hear, and the sound will stop immediately. The goal of this test is to determine the sound intensity that causes discomfort, not to assess whether the sound is loud or soft for you."

Hyperacusis was diagnosed using the criterion proposed by Johnson^(28)^, known as the Johnson Hyperacusis Dynamic Range Quotient (JHQ). This method involves subtracting the pure tone threshold (PTT) from the corresponding Loudness Discomfort Level (LDL) at each frequency (LDL-PTT), yielding the dynamic range. The JHQ is calculated by summing the dynamic ranges across all frequencies and dividing by the number of frequencies tested. Participants with JHQ values below 90 dB were classified as having hyperacusis.

To assess cognitive function, the Mini-Mental State Examination (MMSE) was administered as a cognitive screening tool to control for potential confounding effects on study results. The maximum MMSE score is 30 points, with higher scores indicating better cognitive performance. Due to the well-known influence of education level on the total MMSE scores, this study adopted education-adjusted cut-off scores based on the guidelines proposed by the Brazilian Academy of Neurology^(25)^. Specifically, the cut-off scores were: ≥ 19 points for illiterate individuals; ≥ 24 points for those with 1 to 4 years of education; ≥ 26 points for those with 5 to 8 years; ≥ 27 points for individuals with 9 to 11 years; and ≥ 28 points for individuals with more than 12 years of education.

Data from the T-V1 and T-V2 phases were analyzed using the Statistical Package for the Social Sciences (SPSS), version 23.0. For quantitative variables, measures of central tendency (mean, median), variability (standard deviation), and position (minimum and maximum) were calculated. For qualitative variables, absolute and relative frequencies were determined.

## RESULTS

During the pre-final version testing phase (T-V1), the questionnaire was administered to 30 individuals with hyperacusis, 26 of whom were female (86.6%) and four male (13.4%), aged between 20 and 65 years (mean age of 44 years and 5 months). The adjusted pre-final version (T-V2) was tested on another 30 participants with hyperacusis, of whom 24 were female (80%) and six male (20%), aged between 20 and 69 years, with a mean age of 36 years and 5 months. Table 1 presents the sample characterization by sex and age for stages T-V1 and T-V2.

**Table 1:** Characterization of the sample regarding age in the T-V1 and T-V2 stages

Gender	Stage T-V1						Stage T-V2					
	n	Mean (year)	SD	Median (year)	Min	Max	n	Mean (year)	SD	Median (year)	Min	Max
Female	26	44.0	14.8	48	20	65	24	37.4	18.4	35.5	20	69
Male	4	47.7	9.6	49	33	60	6	43.3	12.7	46.0	26	58
Total	30	44.5	14.3	48	20	65	30	38.6	15.9	36.5	20	69

**Caption:** T-V1 = pre-final version test; T-V2 = adjusted pre-final version test; n = number of participants; SD = standard deviation; Min = minimum value found; Max = maximum value found

The Mini-Mental State Examination (MMSE) is a cognitive screening test applied in this study to identify the risk of dementia and to exclude its potential influence on the results. Given the well-documented effect of education level on MMSE total scores, different cutoff scores were used based on participants’ educational backgrounds. The sample from stages T-V1 and stage T-V2 were found to be homogeneous in terms of total MMSE scores, as shown in Table 2.

**Table 2:** Descriptive statistics of Mini-Mental State Examination scores for the T-V1 and T-V2 stages

**Descriptive Statistics**	**Stage**	
	**T-V1**	**T-V2**
n	30	30
Minimum	25	28
Maximum	30	30
Mean	29,0	29,1
1st Quartile	28	28
Median	29	29
3rd Quartile	30	30

**Caption:** T-V1 = pre-final version test; T-V2 = adjusted pre-final version test; n = number of participants

None of the 30 participants in the T-V2 phase reported difficulties understanding the questionnaire items. All questions were deemed effective, with no discrepancies and a comprehension rate of 100%. Thus, the adjusted version was validated as the culturally adapted version for Brazilian Portuguese (Appendix A). The item comprehension rates in each phase are presented in Table 3.

**Table 3:** Frequency distribution and percentage of item comprehension of the Hyperacusis Handicap Questionnaire

**Questions**	**T-V1 (n=30)**		**T-V2 (n=30)**	
	**Frequency**	**Percentage**	**Frequency**	**Percentage**
**Q1**	26	86.6	30	100.0
**Q2**	30	100.0	30	100.0
**Q3**	30	100.0	30	100.0
**Q4**	25	83.3	30	100.0
**Q5**	30	100.0	30	100.0
**Q6**	30	100.0	30	100.0
**Q7**	30	100.0	30	100.0
**Q8**	27	90.0	30	100.0
**Q9**	30	100.0	30	100.0
**Q10**	30	100.0	30	100.0
**Q11**	30	100.0	30	100.0
**Q12**	26	86.6	30	100.0
**Q13**	30	100.0	30	100.0
**Q14**	30	100.0	30	100.0
**Q15**	30	100.0	30	100.0
**Q16**	30	100.0	30	100.0
**Q17**	30	100.0	30	100.0
**Q18**	30	100.0	30	100.0
**Q19**	30	100.0	30	100.0
**Q20**	30	100.0	30	100.0
**Q21**	30	100.0	30	100.0

**Caption:** T-V1 = pre-final version test; T-V2 = adjusted pre-final version test; n = number of participants

The original version of the HHQ did not include a formal statement to introduce the questions comprising the instrument. Based on the consensus reached by the expert committee during the fourth stage of this study, an introductory statement with instructions for completing the HHQ-BP version was added.

## DISCUSSION

Cultural adaptation is an essential process aimed at adjusting the components of an instrument, such as questionnaires or tests, to ensure their effectiveness in populations with different languages or cultures. This process goes beyond literal translation, involving the analysis and resolution of sociocultural discrepancies that could impact the comprehension and relevance of the instrument’s items. Only after completing cultural adaptation can the instrument be confidently used in a new linguistic and cultural context. This step is critical to ensure that conclusions drawn from the instrument are accurate and applicable to the new cultural setting.

The methodology employed for translating and culturally adapting the HHQ followed the criteria established by COSMIN^(22)^. This initiative, led by an international, multidisciplinary team of researchers with expertise in epidemiology, psychometrics, qualitative research, and healthcare, provides rigorous guidelines for developing and evaluating outcome measurement instruments. The systematic and detailed approach to translating and culturally adapting the questionnaire proved essential for identifying and addressing equivalence-related challenges, offering a more robust method than simple translation and back-translation. In this study, the adaptation process demonstrated a satisfactory level of semantic equivalence between the Portuguese and English versions.

During the linguistic equivalence assessment, the option “not applicable” was included for each questionnaire item, although none of the questions were considered inappropriate for the Brazilian version. The translation and cultural adaptation to Brazilian Portuguese were relatively straightforward due to the clarity and accessibility of the original HHQ version.

The sample in this study differs from that of the original version of the HHQ, particularly regarding participants' sex and age. These differences may be attributed to contextual and sociocultural factors related to the profile of the Brazilian population, as well as the availability and willingness of individuals to participate in the research. The predominance of female participants reflects a common trend in voluntary studies, especially those related to health and behavior. The broader age range was intentionally adopted to better represent the age diversity of the local population and to promote a more inclusive application of the instrument, while still complying with the previously defined inclusion criteria.

While the study successfully achieved the cultural adaptation of the HHQ-BP, it is important to acknowledge certain limitations. Cultural adaptation represents only the initial stage in the questionnaire validation process, and subsequent steps are essential to ensure the reliability and validity of the instrument in Brazilian Portuguese^(29)^. This study did not include the psychometric validation of the HHQ-BP, a limitation currently being addressed in future research conducted by the authors. Consequently, the use of the HHQ-BP should be supplemented by evidence of validity and reliability, which will be obtained in subsequent validation phases.

The study underscored significant advancements in the cultural adaptation process of the HHQ to Brazilian Portuguese (HHQ-BP). The adapted version proved easy to administer and met cultural, semantic, and item equivalence criteria, ensuring that the instrument retained its original meaning. Adding a formal introductory statement for the questions, absent in the original version, was a significant improvement, enhancing clarity and objectivity for Brazilian participants. This modification, stemming from expert committee consensus, reflects the methodological rigor applied during cultural adaptation, laying a solid foundation for the instrument’s upcoming validation phases.

## CONCLUSION

The HHQ was translated and culturally adapted into Brazilian Portuguese following standardized recommendations and guidelines, ensuring cultural and semantic equivalence. The cultural adaptation of the Hyperacusis Handicap Questionnaire (HHQ) to Brazilian Portuguese has been completed, resulting in the final version titled Hyperacusis Handicap Questionnaire – Brazilian Portuguese (HHQ-BP). Validation of this version is currently in progress.

## Appendix A Hyperacusis Handicap Questionnaire in Brazilian Portuguese (HHQ-BP)

Por favor, marque um X para cada questão abaixo. Você deverá responder **Nunca**, **Às vezes** ou **Sempre,** de acordo com o desconforto gerado pela hiperacusia.

**Table:**

		Nunca	Às vezes	Sempre
1.	Com que frequência você sente necessidade de tapar os seus ouvidos para determinados sons ou para reduzir o volume do som?			
2.	Com que frequência você sente desconforto para ler ou realizar atividades em lugares barulhentos?			
3.	Com que frequência você tem dificuldades para se concentrar em alguma tarefa por causa de intolerância ao som?			
4.	Com que frequência você acha que a sua rotina e o seu desempenho no trabalho sofrem interferências negativas devido à intolerância ao som?			
5.	Com que frequência você sente que não consegue apreciar uma música por causa da intolerância ao som?			
6.	Com que frequência você acha difícil escutar por muito tempo quando você está cercado por muitos outros sons no ambiente?			
7.	Com que frequência você sente dificuldade para escutar música usando fones de ouvido?			
8.	Com que frequência você fica impaciente quando conversa em um ambiente barulhento?			
9.	Com que frequência você sente que certos sons o incomodam mais ou trazem dificuldades enquanto você conversa?			
10.	Com que frequência você evita fazer certas atividades ou sair de casa porque você precisa estar em uma situação/lugar barulhento?			
11.	Alguma vez você já se sentiu isolado em um grupo de pessoas devido à intolerância ao som (por exemplo, em festas)?			
12.	Com que frequência as pessoas dizem que você não consegue tolerar sons ou que sua tolerância para certos tipos de som é muito baixa?			
13.	Com que frequência você prefere ficar dentro de casa porque você imagina que irá enfrentar sons altos fora de casa?			
14.	Com que frequência você teve vontade de mudar de local de trabalho por causa de som excessivo?			
15.	Com que frequência você se sente triste por não conseguir tolerar certos sons, como o barulho do trânsito?			
16.	Com que frequência você sente que um lugar barulhento traz estresse e irritação?			
17.	Com que frequência você sente raiva quando está cercado por sons?			
18.	Com que frequência você tem medo de ouvir algum som em particular devido à intensidade desse som?			
19.	Com que frequência você enfrenta problemas emocionais por causa da intolerância ao som?			
20.	Com que frequência você se sente incomodado devido ao fato de que a intolerância ao som está afetando o seu relacionamento com a sua família e os seus amigos?			
21.	Com que frequência você se irrita por causa dos sons?			
**Pontuação total**				

As questões 1 a 7 estão relacionadas ao Handicap funcional; 8 a 14 estão relacionadas ao Handicap social; e 15 a 21 ao Handicap emocional do paciente. As sub-pontuações para cada domínio também podem ser obtidas somando-se as pontuações das respectivas questões de cada categoria

**Table:** PONTUAÇÕES:

Funcional	Social	Emocional	Total
			
